# Compliance with a WHO guideline for cardiovascular disease prevention. A population based cross-sectional study in Argentina: CVD prevention recommendations

**DOI:** 10.31053/1853.0605.v81.n1.41876

**Published:** 2024-03-27

**Authors:** Pablo Gulayin, Laura Gutierrez, Fernando Lanas, Nora Mores, Jacqueline Ponzo, Matías Calandrelli, Rosana Poggio, Vilma Irazola

**Affiliations:** 1 Instituto de Efectividad Clínica y Sanitaria Buenos Aires Argentina; 2 Facultad de Ciencias Médicas, Universidad Nacional de La Plata La Plata Argentina; 3 IGES, Universidad de La Frontera (UFRO) Temuco Chile; 4 Municipalidad de Marcos Paz Marcos Paz Buenos Aires Argentina; 5 Facultad de Medicina, Universidad de la República Montevideo Uruguay; 6 Sanatorio San Carlos Bariloche Río Negro Argentina; 7 Comisión Nacional de Investigaciones Científico Tecnológicas

**Keywords:** guía de práctica clínica, prevención de enfermedades, factores de riesgo de enfermedad cardiaca, practice guideline, disease prevention, heart disease risk factors, guia de prática clínica, prevenção de doenças, fatores de risco de doenças dardíacas

## Abstract

Introduction: Current recommendations for cardiovascular disease (CVD) prevention are guided by CVD risk classification. This study aims to analyze the level of compliance with pharmacological and non-pharmacological recommendations outlined in the World Health Organization (WHO) CVD prevention clinical practice guideline within the general population of two cities in Argentina.

Methods: We analyzed the compliance with preventive recommendations from the WHO 2007 Guidelines for Management of Cardiovascular Risk in two cities of Argentina participating in the CESCAS population-based study in the Southern Cone of Latin America.

Results: 3990 participants were included. Considering the WHO recommendations, the use of antiplatelet medication was 5.6% (95% CI 3.5, 8.9) in primary prevention and 20.5% (95%= CI 16.0, 25.9) in secondary prevention. Regarding lipid-lowering medication, it was 6.7% (CI 95%= 4.4, 10.1) and 15.4% (CI 95%= 11.6, 20.1), respectively. As per non-pharmacological recommendations in the general population: low intake of fruit and vegetables was 78.4% (CI 95%= 76.8, 79.9); low physical activity was 26.9% (CI 95%= 25.3, 28.5), current cigarette smoking was 28.3% (CI 95%= 26.6, 30.0), overweight/obesity was 73.9% (CI 95%= 72.3, 75.6), and excessive alcohol intake was 2.6% (CI 95%= 2.1, 3.4).

Conclusions: It was observed a significantly low compliance with pharmacological and non-pharmacological recommendations for CVD prevention in the general population of two cities in Argentina. Urgent efforts are needed to improve compliance to cardiovascular preventive recommendations promoted by CPG, especially in Low- and Middle- Income Countries.

## Resumen

Introducción: las recomendaciones actuales para la prevención de enfermedades cardiovasculares (ECV) están orientadas por la clasificación del riesgo de ECV. Este estudio tiene como objetivo analizar el nivel de cumplimiento con las recomendaciones farmacológicas y no farmacológicas delineadas en una guía de práctica clínica (GPC) de prevención de la ECV de la Organización Mundial de la Salud (OMS) en población general de dos ciudades de Argentina

Métodos: Se evaluó el cumplimiento de las recomendaciones preventivas de la Guía para el Manejo del Riesgo Cardiovascular de la OMS 2007 en el estudio con base poblacional en el Cono Sur de Latinoamérica CESCAS

Resultados: Se incluyeron 3990 participantes. Considerando las recomendaciones farmacológicas de la OMS, el uso de antiagregantes plaquetarios en prevención primaria fue del 5.6% (IC 95%= 3.5, 8.9) y en prevención secudnaria del 20.5% (IC 95% 16.0, 25.9). En cuanto a la medicación hipolipemiante fue del 6.7% (IC95%= 4.4, 10.1) y del 15.4% (IC 95%= 11.6, 20.1), respectivamente. Cumplimiento de las recomendaciones no farmacológicas en la población general: la baja ingesta de frutas y verduras fue del 78.4% (IC 95%= 76.8, 79.9); baja actividad física del 26,9% (IC 95%= 25.3, 28.5), tabaquismo actual fue del 28.3% (IC 95%= 26.6, 30.0), sobrepeso/obesidad del 73,9% (IC 95%= 72.3, 75.6) y el consumo excesivo de alcohol 2.6% (IC 95%= 2.1, 3.4).

Conclusiones: Se observó un cumplimiento significativamente bajo a las recomendaciones farmacológicas y no farmacológicas para la prevención de ECV en población general de dos ciudades de Argentina. Se necesitan esfuerzos urgentes para mejorar el cumplimiento de las recomendaciones en prevención cardiovascular promovidas por las GPC, especialmente en los países de bajos y medianos ingresos.

## Resumo

Introdução: As recomendações atuais para a prevenção de doenças cardiovasculares (DCV) são orientadas pela classificação de risco de DCV. Este estudo tem como objetivo analisar o grau de cumprimiento das recomendações farmacológicas e não farmacológicas de uma diretriz de prática clínica de prevenção de DCV da Organização Mundial da Saúde (OMS) na população geral de duas cidades da Argentina.

Métodos: Analisamos o cumprimiento das recomendações preventivas das Diretrizes de 2007 para o manejo do Risco Cardiovascular da OMS em duas cidades da Argentina que participaram do estudo populacional CESCAS no Cone Sul da América Latina.

Resultados: Foram incluídos 3990 participantes. Considerando as recomendações da OMS, o uso de medicamentos antiplaquetários foi de 5.6% (IC 95%: 3.5, 8.9) na prevenção primária e de 20.5% (IC 95%: 16.0, 25.9) na prevenção secundária. Em relação à medicação hipolipemiante, foi de 6.7% (IC 95%: 4.4, 10.1) e 15,4% (IC 95%: 11.6, 20.1), respectivamente. Quanto às recomendações não farmacológicas na população geral: baixa ingestão de frutas e vegetais foi de 78.4% (IC 95%: 76.8, 79.9); baixa atividade física foi de 26.9% (IC 95%: 25.3, 28.5), tabagismo atual foi de 28.3% (IC 95%: 26.6, 30.0), sobrepeso/obesidade foi de 73.9% (IC 95%: 72.3, 75.6) e consumo excessivo de álcool foi de 2.6% (IC 95%: 2.1, 3.4).

Conclusões: Observou-se un cumprimento significativamente baixa das recomendações farmacológicas e não farmacológicas para a prevenção de DCV na população geral de duas cidades da Argentina. Esforços urgentes são necessários para melhorar o cumprimento de recomendações preventivas cardiovasculares promovidas pelas diretrizes de prática clínica, especialmente em países de baixa e média renda.

## Introduction

It has been estimated that approximately 80% of annual deaths caused by cardiovascular disease (CVD) occur in low- and middle- income countries (LMICs) ^1^ , being ischemic heart disease and stroke the most important sources of this burden of disease ^2^ . However, despite wide sources of scientific and economic evidence highlighting the benefits preventive medications for CVD, it is considered that small proportion of individuals who should be receiving these medications are properly treated in most LMICs, particularly in secondary prevention ^3, 4^ .

Recommendations based on evidence-based clinical practice guidelines (CPG) aim to reduce fatal and non-fatal cardiovascular disease (CVD) events within the population. These recommendations involve an initial assessment of CVD risk classification, followed by the implementation of more aggressive preventive interventions in higher-risk sub-groups ^5-^ . Based on the 2007 World Health Organization (WHO) recommendations ^5^ , the National Ministry of Health in Argentina published a condensed version for the Assessment and Management of Cardiovascular Risk, specifically tailored for the Prevention of Cardiovascular Disease ^8^ . This guideline incorporated the CVD risk classification chart for Region B of The Americas, and provided pharmacological and non-pharmacological recommendations based on the risk strata for primary or secondary prevention. The development and publication of this guideline were accompanied by various dissemination activities, including cardiovascular management training sessions for the public primary care health team in Argentina. Using population-based data, this study aims to analyze the level of compliance with pharmacological and non-pharmacological recommendations outlined in the WHO CVD prevention clinical practice guideline within the general population of two cities in Argentina.

## Methods

### Study population

The design and sampling method of the CESCAS I study have been previously described ^9, 10^ . Briefly, this is a population-based study that employed a multistage probabilistic sampling approach in the general population of four small to mid-sized cities in the Southern Cone of Latin America: two in Argentina (Bariloche and Marcos Paz), one in Chile (Temuco), and one in Uruguay (Pando-Barros Blancos). The main objective of CESCAS I was to investigate CVD and its risk factors in the general population of the region. A total of 7524 participants, aged 35 to 74 years, were recruited between February 2010 and December 2011. The study adhered to the principles outlined in the Declaration of Helsinki and received approval from the Institutional Review Boards (IRBs) of all participating institutions in Argentina, Chile, Uruguay, and the USA. Written informed consent was obtained from all study participants. For the purpose of the present study, only data from the two cities in Argentina were analyzed.

### Data collection

The data collection process consisted of a home visit and a clinical visit conducted by trained interviewers. The at-home survey collected information on demographic characteristics, health habits, personal history of cardiovascular disease (CVD), CVD risk factors, and medication use. Medication data were collected during the in-home interview through direct observation, and for analysis purposes, all drugs were coded using WHO ATC codes ^11^ . Blood pressure and anthropometric measurements were obtained following standardized protocols and techniques. Three blood pressure (BP) readings were taken with the participant seated after 5 minutes of rest, using a standard mercury or aneroid sphygmomanometer. The mean of the three readings was used for analysis. Weight was measured on standing scales, with the participant wearing light indoor clothing and no shoes, and was recorded in kilograms to one decimal place. Height was measured using a wall-mounted stadiometer, with participants standing without shoes, and was recorded in centimeters to one decimal place. Waist circumference was measured in centimeters to one decimal place, taken 1 cm above the navel at minimal respiration. Overnight fasting blood samples were collected to measure lipids, creatinine, and glucose. The duration of fasting was confirmed before obtaining the blood specimen. Participants who had not fasted for at least 10 hours did not undergo blood collection. Standard methods were used to measure blood glucose, total cholesterol, high-density lipoprotein cholesterol (HDL-c), and triglycerides. The concentration of low-density lipoprotein cholesterol (LDL-c) was calculated using the Friedewald equation for participants with triglyceride levels <400mg/dL.

### Definitions

History of CVD disease for primary/secondary prevention classification was considered in case of self-reported history of coronary disease (angina, myocardial infarction, or coronary revascularization) or cerebrovascular disease (transient ischemic attack, stroke or carotid revascularization). In primary prevention, participants were classified following the Region B of The Americas WHO/International Society of Hypertension (ISH) risk prediction charts ^8^ . The analysis utilized three 10-year CVD risk categories: low (< 10%), moderate (10 to < 20%), and high (≥ 20%), based on sex, age, total cholesterol, blood pressure, smoking status, and diabetes ^8^ .

The CVD preventive recommendations analyzed were based on the "*Prevention of Cardiovascular Disease - Guidelines for assessment and management of cardiovascular risk*" published by WHO in 2007 and the local version by the National Ministry of Health in Argentina in 2009 ^5, 8^ . To evaluate how much pharmacological preventive recommendations were followed in the general population, indications for the use of aspirin and statins were considered. These guidelines recommended the use of aspirin in case of secondary prevention and primary prevention with 10-years CVD risk ≥ 20%. Statin use was recommended for secondary prevention and for primary prevention in these situations: 1) individuals with total cholesterol (TC) ≥310 mg/dL, 2) 10-years CVD risk ≥ 20%, age >40 years and TC >190 mg/dL and/or LDL-c >114 mg/dL or 3) 10-years CVD risk ≥ 30%. Considering the possible use of alternative preventive drugs, a comprehensive analysis was conducted considering 'antiplatelet medication' (aspirin or clopidogrel) and 'lipid-lowering medication' (statins or ezetimibe). Non-pharmacological recommendations from the CPG considered for the analysis were: 1) do not smoke, 2) eat at least 400 g a day (~ 5 servings) of fruits and vegetables, 3) take at least 30 minutes of moderate physical activity per day (categorized as ≥ 600 MET-minutes/per week), 4) control weight to avoid overweight or obesity, and 5) reduce alcohol consumption to less than 3 units among drinkers.

### Data analysis

To describe the general characteristics and prevalence of cardiometabolic risk factors, all variables were considered as categorical. Results were analyzed by primary or secondary prevention. Proportions were weighted to represent the general adult population aged 35-74 in the study sites, according to the study sampling design. 95% confidence Intervals (95% CI) were calculated using standard errors that take the complex survey design into account. The same approach was applied to analyze the use of medication and compliance of non-pharmacological recommendations by the level of prevention. Finally, to estimate the 10-year CVD risk in primary prevention, we used the WHO/ISH cardiovascular risk scores R package published by Collins et. al ^12^

## Results

**Figure 1:**
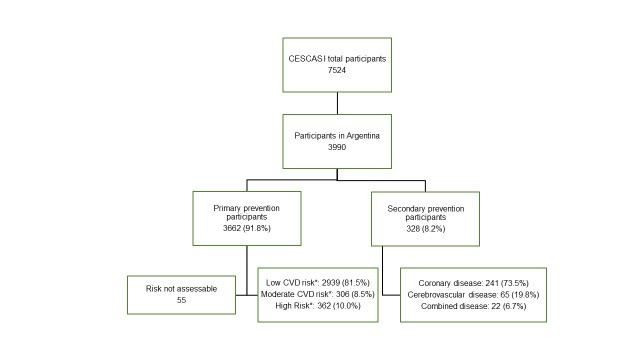
History of CVD and risk strata in the population study *WHO/ISH 10-year CVD risk in primary prevention: low (< 10%), moderate (10 to < 20%), and high (≥ 20%). Secondary prevention: self-reported history of coronary disease (angina, myocardial infarction, or coronary revascularization) or cerebrovascular disease (transient ischemic attack, stroke or carotid revascularization).

Figure 1 displays the distribution of the study population according to the level of CVD prevention. From the CESCAS I study, 3990 participants from Argentina were included in the analysis. In this population, 8.2% reported history of at least one CVD event. Coronary heart disease was the most common reported event, accounting for 73.5%. Concerning the population in primary prevention and their distribution according to the WHO 10-year CVD risk strata, the majority had low risk (81.5%), followed by high risk (10.0%) and moderate risk (8.5%). CVD risk estimation was not possible for 55 participants due to the absence of necessary lab measurements. Overall, individuals with high risk in primary prevention and those in secondary prevention constituted 17.3% of the entire study population.

Table 1 summarizes socio-demographic and CVD risk factors distribution according to the level of prevention. In both groups, primary and secondary prevention, over 50% of the population did not attain a high-school education level. Furthermore, a higher proportion of people reported not having health insurance in the population with a history of CVD (56.2%) compared to the primary prevention group (37.5%). Concerning the presence of CVD risk factors, nearly half of the primary prevention group (48.1%) had central obesity, 42.5% had hypertension, 1.6% had chronic kidney disease, more than half (54.8%) had dyslipidemia, and 8.4% had diabetes. The prevalence of these risk factors was higher in the secondary prevention group, particularly for hypertension (65.8%), dyslipidemia (67.7%), and diabetes (21.5%).

**Table 1:** General characteristics and prevalence of cardiometabolic risk factors by level of cardiovascular prevention

Columna1	**Less than high school level**	**No health insurance**	**Central obesity**	**Hypertension**	**CKD**	**High LDL-c **	**Low HDL-c**	**Hyper-triglyceridemia**	**Dyslipidemia**	**Diabetes**
Primary prevention										
Overall	64.6 (62.7, 66.6)	37.5 (35.6, 39.3)	48.1 (46.2, 50.1)	42.5 (40.6, 44.4)	1.6 (1.2, 2.0)	19.2 (17.7, 20.7)	33.6 (31.7, 35.5)	19.7 (18.1, 21.2)	54.8 (52.8, 56.7)	8.4 (7.4, 9.4)
Men	66.5 (63.5, 69.5)	38.4 (35.4, 41.3)	35.7 (32.8, 38.5)	48.4 (45.3, 51.5)	1.6 (1.0, 2.1)	20.3 (17.8, 22.8)	45.9 (42.8, 49.0)	26.3 (23.5, 29.0)	65.7 (62.8, 68.7)	7.7 (6.3, 9.2)
Women	62.9 (60.5, 65.4)	36.7 (34.4, 39.0)	59.5 (57.0, 61.9)	37.2 (34.9, 39.4)	1.6 (1.1, 2.1)	18.2 (16.4, 19.9)	22.4 (20.4, 24.5)	13.7 (12.1, 15.3)	44.9 (42.4, 47.3)	9.0 (7.7, 10.3)
Age group										
35-44	55.4 (51.6, 59.2)	41.2 (37.5, 44.8)	40.7 (37.0, 44.3)	26.0 (22.6, 29.5)	0.1 (0.0, 0.2)	11.7 (9.2, 14.3)	36.7 (33.0, 40.4)	18.3 (15.3, 21.2)	51.7 (47.9, 55.5)	4.2 ( 2.8, 5.7)
45-54	63.4 (60.1, 66.7)	43.1 (39.7, 46.4)	45.8 (42.4, 49.1)	40.5 (37.2, 43.9)	0.4 ( 0.0, 0.7)	19.3 (16.6, 22.1)	33.3 (30.1, 36.5)	21.5 (18.6, 24.3)	54.2 (50.8, 57.6)	7.1 ( 5.4, 8.8)
55-64	74.3 (71.4, 77.3)	34.4 (31.3, 37.5)	56.8 (53.5, 60.1)	58.1 (54.9, 61.4)	3.6 ( 2.3, 4.8)	26.6 (23.6, 29.6)	32.2 (29.1, 35.4)	20.7 (18.0, 23.5)	59.6 (56.3, 62.9)	14.3 (12.0, 16.6)
65-74	80.1 (76.5, 83.7)	16.7 (13.8, 19.5)	62.7 (58.4, 67.0)	72.5 (68.6, 76.4)	6.1 (4.1, 8.1)	29.2 (25.2, 33.3)	26.8 (22.9, 30.8)	17.5 (14.2, 20.8)	57.6 (53.2, 62.0)	14.2 (11.2, 17.2)
Secondary prevention										
Overall	52.3 (50.9, 53.7)	56.2 (54.8, 57.6)	53.6 (47.2, 60.1)	65.8 (59.7, 72.0)	5.6 (2.4, 8.8)	30.4 (24.4, 36.3)	41.7 (35.2, 48.1)	29.0 (23.0, 35.0)	67.7 (61.7, 73.6)	21.7 (16.6, 26.8)
Men	52.4 (50.3, 54.5)	54.8 (52.7, 56.8)	45.6 (36.8, 54.3)	74.1 (66.2, 81.9)	7.2 ( 2.1, 12.2)	27.5 (19.2, 35.9)	50.8 (41.9, 59.8)	33.0 (24.5, 41.5)	69.9 (61.7, 78.0)	21.2 (14.2, 28.2)
Women	52.2 (50.4, 54.1)	57.4 (55.6, 59.3)	64.3 (55.6, 73.1)	54.8 (45.6, 64.0)	3.5 ( 0.4, 6.6)	34.0 (25.5, 42.4)	29.8 (21.1, 38.5)	23.8 (15.6, 32.0)	64.8 (56.0, 73.5)	22.4 (15.0, 29.8)
Age group										
35-44	40.8 (38.2, 43.4)	56.7 (54.0, 59.5)	45.9 (26.8, 65.0)	51.6 (32.4, 70.8)	5.8 (0.0, 16.7)	10.1 (0.0, 22.8)	54.0 (34.6, 73.5)	46.0 (26.4, 65.5)	72.6 (55.3, 90.0)	10.5 (0.0, 24.3)
45-54	52.0 (49.5, 54.5)	59.3 (56.9, 61.7)	47.0 (33.2, 60.7)	54.7 (40.9, 68.4)	5.3 (0.0, 11.9)	32.6 (19.0, 46.1)	41.8 (28.0, 55.7)	24.5 (12.5, 36.5)	64.1 (51.0, 77.3)	10.3 ( 2.7, 17.9)
55-64	61.6 (59.2, 64.0)	54.5 (52.1, 56.9)	56.7 (46.5, 66.8)	69.6 (60.0, 79.2)	5.5 ( 0.8, 10.2)	32.7 (22.8, 42.5)	41.4 (31.3, 51.5)	32.1 (22.5, 41.7)	67.2 (57.2, 77.1)	31.0 (21.5, 40.6)
65-74	71.9 (69.4, 74.4)	50.1 (47.4, 52.9)	61.3 (51.0, 71.7)	81.1 (73.2, 88.9)	5.9 ( 1.0, 10.7)	38.9 (28.4, 49.3)	34.0 (23.8, 44.2)	19.0 (10.9, 27.1)	68.3 (58.7, 77.9)	28.9 (19.3, 38.5)

Central obesity: waist circumference ≥102 cm for men and ≥88 cm for women

Hypertension: systolic blood pressure ≥140 mm Hg and/or diastolic blood pressure ≥90 mm Hg and/or use of antihypertensive medication

CKD (Chronic kidney disease): estimated-glomerular filtration rate based on CKD-EPI equation <60 ml/min/173 m2

High LDL-cholesterol: LDL-cholesterol ≥160 mg/dL and/or use of lipid-lowering medication; Low HDL-cholesterol: HDL-cholesterol < 40 mg/Dl

Hypertriglyceridemia: triglyceride ≥200 mg/dL

Dyslipidemia: total cholesterol ≥240mg/dL and/or LDL-cholesterol ≥160mg/dL and/or HDL-cholesterol <40mg/dL and/or triglyceride ≥200mg/dL and/or use of lipid-lowering medication

Diabetes: fasting glucose ≥126mg/dL or self-reported history of diabetes

**Figure 2:**
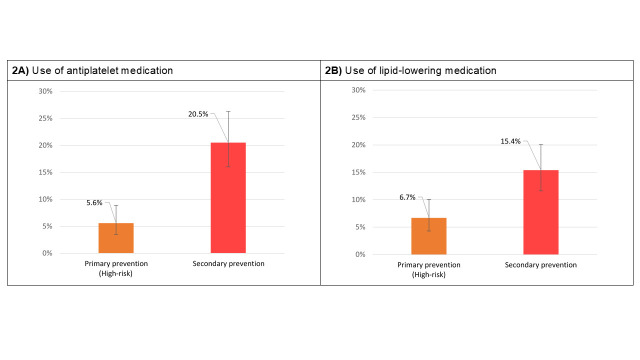
Use of preventive medication by level of prevention Primary prevention (High-risk): WHO/ISH 10-year CVD risk ≥ 20%; Secondary prevention: self-reported history of coronary disease (angina, myocardial infarction, or coronary revascularization) or cerebrovascular disease (transient ischemic attack, stroke or carotid revascularization).

Regarding the use of preventive medication in accordance with guideline recommendations, Figure 2A illustrates the prevalence of antiplatelet medication (aspirin or clopidogrel) use among individuals in secondary prevention and those at high risk in primary prevention. Among individuals using antiplatelet medication, 91.3% were taking aspirin, 5.0% were taking clopidogrel, and 3.7% were on combined therapy. The utilization of antiplatelet medication was significantly higher among individuals in secondary prevention [20.5% (95% CI 16.0, 25.9)] than in primary prevention [5.6% (95% CI 3.5, 8.9)]. When considering the entire population recommended for antiplatelet medication, the overall utilization rate was 13.1% (95% CI 10.5, 16.2). On the other hand, Figure 2Bpresents the utilization of lipid-lowering medication (statins or ezetimibe) in accordance with the recommendations in the WHO guideline. Among all participants using these medications, 93.2% were prescribed statins, 1.2% were taking ezetimibe, and 5.6% were on a combination of both drugs. In this population, 15.4% (95% CI= 11.6, 20.1) of participants in secondary prevention were utilizing lipid-lowering medication, while 6.7% (95% CI= 4.4, 10.1) of individuals in primary prevention were taking statins or ezetimibe. Overall, the utilization rate of this medication, according to the WHO guideline, was 10.9% (95% CI= 8.6, 13.7). Finally, when it as analyzed the use of combined preventive medication in individuals with history of CVD, we found that 8.9% (95% CI= 6.1, 13.1) was using antiplatelet and lipid-lowering medication simultaneously.

Lastly, Table 2 presents the rate of lack of compliance with non-pharmacological recommendations stratified by risk strata. Overall, 78.4% had low intake of fruit and vegetables, 26.9% reported low level of physical activity, 28.3% were current smokers, 73.9% were either overweight or obese, and 2.6% had excessive alcohol intake. These results were similar among all risk strata in primary prevention and secondary prevention, except for low physical activity, which showed a higher prevalence in the population with CVD history (36.5%).

**Table 2:** Lack of follow up of non-pharmacological recommendations by cardiovascular risk strata

	**Global**	**Primary prevention**	**Primary prevention**			**Secondary prevention**
			**Low CVD risk**	**Moderate CVD risk**	**High CVD risk**	
**Low intake of fruit**	78.4 (76.8, 79.9)	78.3 (76.7, 79.8)	78.8 (77.0, 80.5)	72.1 (65.9, 77.5)	78.5 (72.7–83.3)	78.9 (73.1, 83, 6)
**Low physical activity**	26.9 (25.3, 28.5)	26.1 (24.5, 27.8)	24.9 (23.1, 26.7)	31.8 (26.1, 37.9)	35.9 (30.1, 42.2)	36.5 (30.5, 42.5)
**Current cigarrette smoking**	28.3 (26.6, 30.0)	28.3 (26.6, 30.2)	27.6 (25.7, 29.6)	34.6 (28.7, 41)	33.3 (27.3, 39.9)	27.7 (22.2, 34.0)
**Overweight/obesity**	73.9 (72.3, 75.6)	73.5 (71.8, 75.2)	71.7 (70.0, 73.8)	81.2 (75.8, 85.6)	88.0 (78.4, 88.3)	79.8 (74.3, 84.5)
**Excessive alcohol intake**	2.6 (2.1, 3.4)	2.4 (1.8, 3.1)	2.1 (1.5, 2.9)	4.1 (1.9, 8.3)	3.9 (1.9, 7.8)	6.5 (3.7, 11.1)

Values are expressed as weighted proportions (95% confidence interval)

Low fruit and vegetable intake: <5 servings per day; low physical activity: < 600 MET-minutes/per week

Overweight: body-mass index ≥25 and <30 kg/m ^2^

Excessive alcohol intake: 3 or more units of alcohol per day

WHO/ISH 10-year CVD risk in primary prevention: low (< 10%), moderate (10 to < 20%), and high (≥ 20%)

Secondary prevention: self-reported history of coronary disease (angina, myocardial infarction, or coronary revascularization) or cerebrovascular disease (transient ischemic attack, stroke or carotid revascularization)

## Discussion

This study evaluated the extent to which cardiovascular prevention recommendations were followed in the general population of Argentina. The findings revealed a very low rate of adherence to preventive medication, including antiplatelet and lipid-lowering medication, in both primary and secondary prevention. Less than 10% of individuals with high risk in primary prevention and only 1 in 5 participants in secondary prevention were using aspirin or clopidogrel for antiplatelet therapy. Similarly, the utilization of lipid-lowering medication was also poor, with less than 10% of individuals in primary prevention and approximately 15% in secondary prevention following the recommended guidelines. Among individuals in secondary prevention, less than 1 in 10 were adhering to both preventive recommendations simultaneously (antiplatelet and lipid-lowering medication). Additionally, there was a significant lack of compliance with non-pharmacological recommendations. Of particular concern, the inappropriate adherence to these non-pharmacological recommendations was either similar or worse in the high CVD risk and secondary prevention groups compared to the lower risk stratum.

CVD prevention not only aims to reduce new CVD events or CVD-related mortality but also focuses on improving the overall health quality and life expectancy of the target population ^13^ . In recent years, there has been a growing emphasis on the comprehensive assessment of an individual's global CVD risk through the use of classification charts or clinical algorithms. This approach helps to identify population subgroups that would receive a greater benefit from more intense interventions and has been recognized by the WHO as one of the "best buys" for addressing noncommunicable diseases ^14^ .

The insufficient utilization of preventive medication in secondary prevention has been consistently reported in other studies, such as the Prospective Urban Rural Epidemiological (PURE) study ^15^ , the European Action on Secondary and Primary Prevention by Intervention to Reduce Events (EUROASPIRE studies(16,17)) or the WHO study on Prevention of REcurences of Myocardial Infarction and Stroke (WHO-PREMISE) ^4^ . The findings of the present study align more closely with the rates reported in the PURE study, which globally reported a 25.3% use of antiplatelet drugs and a 14.6% use of statins among individuals with self-report history of cardiovascular disease recruited from communities. However, it is important to mention that the participating countries in that study varied in terms of economic development and sociocultural diversity, with significantly higher medication utilization rates observed in high-income countries. In contrast, the EUROASPIRE IV study, a cross-sectional study conducted in 24 European countries to assess the management of patients with a history of coronary heart disease (CHD), reported a much higher utilization rate of preventive medication (93.8% for antiplatelet medication and 85.7% for statins) among patients receiving healthcare at selected hospitals. In addition, in the WHO-PREMISE study conducted among patients with a diagnosis of CHD and cerebrovascular disease in healthcare facilities in low- and middle-income countries, the reported use of aspirin was 81.2% and 70.5%, and for statins, it was 20.8% and 12.2%, respectively.

Regarding primary prevention, although there is an improvement in the utilization of more intensive evidence-based therapies, there still exists a significant gap in the adequate implementation of preventive recommendations in populations without a history of cardiovascular disease (CVD) ^18^ . A cross-sectional study conducted in Spain on a random sample of 2270 individuals receiving primary care described a suboptimal utilization of cardio-protective drugs in primary prevention for patients with a high cardiovascular risk ^19^ . This study examined the appropriate use of drug therapy based on the European Guidelines on Cardiovascular Disease Prevention and employed the Systematic Coronary Risk Evaluation (SCORE) to assess cardiovascular risk. The results indicated that the rate of high-risk individuals receiving statins and antithrombotic medication was 4.5% and 3.4%, respectively. In Argentina, a recent study conducted in the context of more rigorous international recommendations for statin use revealed the existence of clinical variability and controversies among doctors regarding the utilization of statins ^20^ . Furthermore, a recently conducted cluster randomized clinical trial at the public primary care level in Argentina evaluated an educational intervention aimed at improving the utilization of statins ^21^ . The results showed that after one year of follow-up, only 7.7% of participants in the control group who were undergoing statin treatment received an adequate dose ^21^ . Finally, it is important to note that while the recommendation for aspirin use in secondary prevention has been firmly established in clinical guidelines for years, the net benefit of aspirin in primary prevention has been under discussion considering the associated increase in major and minor bleeding events ^22, 23^ . Actually, the national clinical practice guideline on cardiovascular prevention published by Argentina's National Ministry of Health in 2023 discourages the routine use of aspirin in adults without established cardiovascular disease ^24^ .

In addition, our study has identified a low rate of compliance with non-pharmacological interventions across all CVD risk categories. It is known that adherence to lifestyle changes in chronic conditions is poorer compared to pharmacological treatments ^25^ . Many of the rates reported in our study were similar to those documented in EUROASPIRE V for patients in secondary prevention ^17^ or exhibited a similar trend as the rates reported in the 2018 Argentina population-based survey of CVD risk factors ^26^ .

These findings hold significant relevance within the context of new international cardiovascular prevention recommendations and future public health interventions aimed at their dissemination and implementation. Consequently, further studies are necessary to comprehend the factors associated with low compliance to CVD prevention interventions in the general population ^27^ . Three primary concepts pertaining to this issue merit attention: 1) Variations in clinical practice, which result in non-standardized healthcare and non-evidence-based clinical decisions. This can be partially attributed to the challenge of overcoming the inertia of "normal or usual practice," which inadequately aligns with updated guidelines ^28^ ; 2) Inadequate adherence to chronic treatments among patients. Encouraging appropriate adherence to long-term therapies stands as a cornerstone of the Chronic Care Model, with current estimates suggesting adherence rates around 50% among chronic patients and even lower in LMICs ^29^ ; and 3) Ensuring adequate access to effective therapies continues to be a global concern contributing to disparities in cardiovascular healthcare ^3^ . In fact, socio-demographic and clinical characteristics of the population included in this study might have added barriers to appropriate compliance with preventive recommendations, such as the observed low educational level, the significant proportion of people without health insurance, and a high prevalence of risk factors, especially among those with a history of cardiovascular disease that may have led to a high burden of associated comorbidity.

This study assessed the extent to which pharmacological and non-pharmacological recommendations, derived from an already disseminated and implemented CVD prevention guideline, were followed across different CVD risk categories in the general population of Argentina. These results not only highlighted the significant gap between real-world practice and ideal interventions in CVD prevention but also underscored the more pronounced disparity observed in LMICs compared to other regions of the world. However, the following limitations should be noted. The self-reporting of CVD cases may not capture the total number of CVD events due to recall bias or misclassification, although this method has been previously utilized in other community-based studies examining CVD epidemiology or the implementation of CVD recommendations ^15, 30^ . The same limitation applies to medication use reporting; however, this bias could be mitigated as participants were requested to bring the packages of all medications they were taking, allowing for a more accurate assessment of the type and dosage of drugs being used. Finally, although the current analysis is timely since it highlights a low compliance with specific cardiovascular prevention guidelines, it's important to consider that these results might be subject to change over time due to variations in socioeconomic factors in our context. As highlighted by Thomas Frieden, socioeconomic factors (such as poverty and education) are foundational elements of the health impact pyramid, representing the tier with the greatest influence on population health ^31^ . Therefore, these results might have been influenced by the subsequent socioeconomic crisis in Argentina as it previously happened in other moments in the country history ^32^ .

## Conclusion

Significant non-compliance with both pharmacological and non-pharmacological recommendations for CVD prevention was observed, consistent with similar studies conducted in LMICs. Urgent efforts are needed to develop comprehensive interventions aimed at improving the implementation, dissemination, training, and monitoring of CVD prevention recommendations for both primary and secondary prevention. This need is further exacerbated by the global population's increasing CVD risk due to the aging population and the rising prevalence of overweight/obesity, sedentary lifestyles, and diabetes."
